# 2395 Developing a conceptual model of healthcare access for adolescent Latinas in the US South

**DOI:** 10.1017/cts.2018.248

**Published:** 2018-11-21

**Authors:** Mercedes M. M. Aleman, Gwendolyn Ferreti, Isabel C. Scarinci

**Affiliations:** The University of Alabama

## Abstract

OBJECTIVES/SPECIFIC AIMS: Alabama (AL) experienced a 145% increase in its Latino population between 2000 and 2010; making it the state with the second fastest growing Latino population in the United States (US) during that time. Adolescent Latinas in the United States and in AL are disproportionately affected by sexual health disparities as evidenced by the disproportionate burden of HIV, STIs and early pregnancy compared with their non-Hispanic, White counterparts. In 2011, Alabama passed 1 of the harshest anti-immigration laws in the nation. Following the passing of this law, county health department visits among Latino adults decreased by 25% for STIs and 13% for family planning. Empirical data with adult Latinas in the Southeast suggest significant barriers to sexual healthcare access. However, to our knowledge, no other researchers have examined barriers and facilitators to sexual healthcare access for this subpopulation. Therefore, the goal of this 3-phase study is to: (a) better understand the factors underlying sexual health disparities and gaps in healthcare access among adolescent Latinas; (b) develop a conceptual model based on these data and the extant literature summarizing the theorized pathways through which factors at differing levels of the socioecological model of health (SEMH) impact sexual healthcare access for this group; and (c) develop community-driven, theory-based, culturally-relevant, multilevel intervention strategies to reduce sexual health disparities and increase sexual healthcare access for adolescent Latinas through a community-engaged, intervention mapping process. Community based participatory research (CBPR), which ensures equitable participation of stakeholder groups through partnerships, and the SEMH, which conceptualizes the individual as nested within a set of social structures, provide the philosophical and theoretical frameworks for the work. METHODS/STUDY POPULATION: From January of 2017 to December of 2017 we completed phase 1 of the study: conducting and analyzing 20 semi-structured qualitative interviews with adolescents who: self-identified as Latina, were between 15 and 20 years of age, had been in the United States for over 5 years, and lived in one of the counties of West AL and 15 semi-structured qualitative interviews with key stakeholders (healthcare providers, parents, policy makers, etc.) who regularly work with Latina adolescents. Interview participants were recruited through purposeful-convenience sampling. Two bilingual (in English and Spanish) coders used an iterative process (between independent coding and consensus building) to analyze the data using NVivo 11. Phase 2 of the study is currently underway: constructing a conceptual model on sexual healthcare access for young Latinas in Alabama. We have utilized an iterative process between qualitative interview data collected in phase 1 and review of the extant literature to draft a conceptual model of healthcare access among adolescent Latinas in the US South. This model will serve as the foundation of future studies including the development of intervention strategies through a CBPR process (phase 3), to commence in January 2018. RESULTS/ANTICIPATED RESULTS: PHASE 1: Several barriers and facilitators to sexual healthcare access emerged from the semi-structured qualitative research interviews with young women. These included: (1) parental approval/disapproval and embarrassment (“pena”); (2) structural barriers/facilitators to care (e.g., lack of transportation, flexible clinic hours); and (3) negative/positive experiences with providers (e.g., perceived discrimination based on immigrant status). Key stakeholders identified the following barriers and facilitators to sexual healthcare access for adolescent Latinas in their interviews: (1) language barriers/need for interpreters and outreach workers to work with young Latina women; (2) need for better sexual health education across the state; (3) lack of knowledge among young women and their parents about institutions in general and sexual healthcare, in specific; and (4) perceived lack of “deservingness” and discrimination from providers/“not my patients” phenomenon. PHASE 2: This presentation will summarize the development of our conceptual model (see drafts attached). For ease of interpretation, we have created 2 sub-models (centering gender and immigration, respectively) which summarize theorized pathways through which policy, community, organizational, and family-level factors influence young Latina women’s access to sexual healthcare services. DISCUSSION/SIGNIFICANCE OF IMPACT: The proposed research is significant because: (1) the state of AL experienced a dramatic increase in its Latino/a population over the last 15 years and adolescent Latinas in AL are disproportionately affected by sexual health disparities; (2) to our knowledge, this is the only study to examine the multilevel factors associated with sexual healthcare access for adolescent Latinas in the South and inform intervention strategies to promote sexual healthcare access in this population; (3) the work is being conducted under the philosophical lens of CBPR such that community members are involved in every step of the research process, resulting in culturally relevant and youth-specific intervention strategies.

